# Evidence for Anti-*Pseudogymnoascus destructans* (*Pd*) Activity of Propolis

**DOI:** 10.3390/antibiotics7010002

**Published:** 2017-12-21

**Authors:** Soumya Ghosh, Robyn McArthur, Zhi Chao Guo, Rory McKerchar, Kingsley Donkor, Jianping Xu, Naowarat Cheeptham

**Affiliations:** 1 Department of Biological Sciences, Thompson Rivers University, Kamloops, BC V2C 0C8, Canada; sghosh@tru.ca (S.G.); rmcarthur645@gmail.com (R.M.); michelle8472@hotmail.com (Z.C.G.); rory-mckerchar@hotmail.com (R.M.); kdonkor@tru.ca (K.D.); 2 Department of Biology, McMaster University, Hamilton, ON L8S 4K1, Canada; jpxu@mcmaster.ca

**Keywords:** propolis, white-nose syndrome, *Pseudogymnoascus destructans*, anti-*Pd* activities, anti-fungal, fungal infection in bats

## Abstract

White-nose syndrome (WNS) in bats, caused by *Pseudogymnoascus destructans* (*Pd*), is a cutaneous infection that has devastated North American bat populations since 2007. At present, there is no effective method for controlling this disease. Here, we evaluated the effect of propolis against *Pd* in vitro. Using Sabouraud dextrose agar (SDA) medium, approximately 1.7 × 10^7^ conidia spores of the *Pd* strain M3906-2/mL were spread on each plate and grown to form a consistent lawn. A Kirby–Bauer disk diffusion assay was employed using different concentrations of propolis (1%, 2%, 3%, 4%, 5%, 10%, 15%, 20%, 25%), in plates incubated at 8 °C and 15 °C. At 8 °C and 15 °C, as the concentration of propolis increased, there was an increasing zone of inhibition (ZOI), reaching the highest degree at 10% and 25% concentrations, respectively. A germule suppression assay showed a similar effect on *Pd* conidia germination. A MALDI-TOF-MS analysis of propolis revealed multiple constituents with a potential anti-*Pd* activity, including cinnamic acid, p-coumaric acid, and dihydrochalcones, which could be further tested for their individual effects. Our study suggests that propolis or its individual constituents might be suitable products against *Pd*.

## 1. Introduction

White-nose syndrome (WNS) has devastated many eastern North American bat populations since 2007, killing more than six million bats [1]. Since the first observations of mortality at a cave near Albany, New York in 2007, WNS has spread to 31 US states and 5 eastern Canadian provinces [2], most recently appearing in 2017 in the states of Mississippi, Texas, and Washington, USA [2]. The etiological agent of WNS is *Pseudogymnoascus destructans* (*Pd*), a psychrophilic fungus that grows optimally on hibernating bats at temperatures between 12–16 °C [3].

*Pseudogymnoascus destructans (Pd)* preferentially infects thinly haired regions on the skin of hibernating bats, and is able to degrade collagen and invade living tissues [4]. Hibernating bats lower their body temperature to near-ambient temperature during torpor bouts [5]. During hibernation, bats generally seek microclimates that remain above freezing and can be as warm as 15 °C or more for some species [5,6,7], and are relatively high in humidity. Bats generally adapt to the temperature of their surroundings to maximize their energy budget [8]. The similarity between the preferred hibernating environment of bats and the optimal growth condition of the *Pd* pathogen is a major contributor to the WNS epidemic. *Pd* infection disrupts the normal torpor and arousal cycles of hibernating bats [9], causing premature depletion of fat reserves, in addition to electrolyte imbalances and dehydration, resulting in mortality [10]. Bats are essential components of both the natural, agricultural, and other human ecosystems [11]. They play important roles in maintaining ecosystem stability, consume insects that are human or animal pests, and redistribute nutrients through their guano [11,12]. To reduce bat mortality and eliminate the likelihood of species extinction by *Pd*, it is essential to identify effective methods to control *Pd*.

In regard to investigations of anti-*Pd* agents, several recent studies have identified putative anti-*Pd* agents, including (i) volatile compounds produced by the bacteria *Rhodococcus rhodochrous* DAP96253 [13] and by *Pseudomonas spp.* isolated from bat wings [14]; (ii) cold-pressed, terpeneless orange oil (CPT) [15]; and (iii) sesquiterpene trans, trans-farnesol (*Candida albicans* quorum-sensing compound) [13]. Our goal was to identify alternative or additional potent treatments against *Pd* using substances likely to be unharmful to cave environments and to bats.

Propolis is a resinous substance produced by honey bees in beehives throughout the year [16,17]. Stingless bees are widely spread, especially in the tropical and subtropical areas of the world. Propolis produced from such bees possesses therapeutic properties [18], including antimicrobial, antitumor [18], antioxidant [19], anti-stimulant [20], anti-inflammatory [21,22,23,24], antiulcer [22,23,24,25], and anti-HIV activities [25]. For instance, two compounds, cardanol and cardol, isolated from a Thai propolis, possessed antiproliferation and cytotoxicity against carcinomas originated from the lungs, the liver, and the colon [26]. Khacha-ananda et al. 2016 [19] found that ethanolic extracts of propolis (EEP) obtained from Chiang Mai, Thailand, exhibited higher antioxidant activity than EEP from other sources. In 2005, Hu et al. [23] showed that both the ethanol and the water extracts of propolis had anti-inflammatory activities in mice and rats [21]. Significant anti-HIV activities [EC(50) < 0.1 µg/mL, TI > 186] resulted from moronic acid (triterpenoids) isolated from a Brazilian propolis [25]. Additionally, because of its antiviral, antibacterial, and antifungal activities, propolis has been used in human healthcare to treat colds, wounds, ulcers, and rheumatism [27,28]. Propolis was found to exhibit antagonistic effects against a number of Gram-positive cocci and rods [29]. A recent study by Shimizu et al. 2011 [30], showed that the ethanol extract of propolis from Brazil had antiviral activities. When administered orally or cutaneously to herpes simplex virus type 1 (HSV-1)-infected mice, the ethanol extract of propolis significantly reduced the herpetic skin lesions and enhanced delayed-type hypersensitivity [30]. Silici et al. 2006 [31] reported that propolis had antifungal activities against 15 strains belonging to four species of yeasts isolated from patients with superficial mycoses.

Traditionally, studies on the medicinal benefits of propolis have attributed its effects to its complex composition and to the synergistic effects among its complex chemical constituents [32,33]. Another emerging theme is that the chemical composition of propolis is highly dependent on the geographical location, botanical origin [34], and bee species [35]. In different ecosystems, there are different plant species, and these plants can vary in their secretion and exudates, and therefore provide diverse food sources to bees [16]. Thus, the variability in chemical composition among propolis from different sources can be large. For example, propolis produced in the Pacific region contains geranyl flavanones which are also a typical component for the African propolis [17]. The green propolis of Brazil has prenylated phenylpropanoids (e.g., artepillin C) and diterpenes as major components [17], while the propolis of temperate regions consists of flavonoids lacking the B-ring substituents, namely, chrysin, galangin, pinocembrin, pinobanksin, caffeic acid phenethyl ester [36]. These unique mixtures of constituents from different sources likely contribute to the observed multiple effects including not only the broad biological effects described above, but also the inhibition of nuclear factor κ-B, cell proliferation, cell arrest, and apoptosis [17,36].

In this study, we tested the effectiveness of the commercially available propolis purchased from Natural Factors in Coquitlam, British Columbia, Canada on the WNS agent *Pd*. We investigated the in vitro antagonistic properties of propolis against *Pd*. We also tested the inhibition of germule development when incubated with propolis. This study is the first investigation of the anti-*Pd* activities of propolis.

## 2. Results

### 2.1. Kirby–Bauer Diffusion Assay

**Figure 1:**
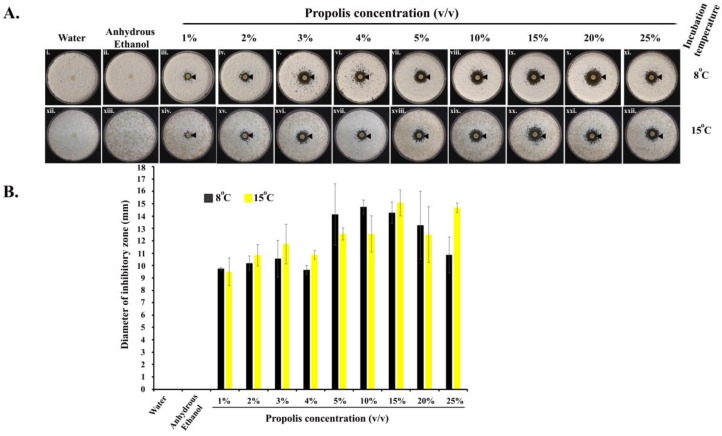
Anti-*Pd* activity of propolis. (**A**) Images (i–xi) and (xii–xxii) indicate the activity of propolis at 8 °C and 15 °C, respectively. The black arrowheads indicate the zone of inhibition of *Pd* when treated with different concentrations of propolis in comparison to water and anhydrous ethanol treatments; (**B**) diameter of the zones of inhibition at 8 °C and 15 °C. The error bars are standard deviations of the diameters.

Propolis exhibited anti-*Pd* activities at all the concentrations (1–25%) we tested (in quadruplets), as revealed by a clear ‘zone of inhibition’ around each of the impregnated paper discs, in comparison to discs treated with water or anhydrous ethanol (Figure 1(Ai,ii,xii,xiii)). ANOVA tests showed that the different concentrations of propolis differed significantly in their inhibitory effects (Figure 1(Aiii–xi,xiv–xxii)). Propolis significantly inhibited the growth of *Pd* at 8 °C (one-way ANOVA; F = 8.309; df = 8; *p* = 0.100^−4^) as well as at 15 °C (one-way ANOVA; F = 8.704; df = 8; *p* = 0.839^−5^). Along the concentration gradient, at 8 °C, the diameter of the inhibitory zones initially increased with the increase in propolis concentration, reaching the highest value at the propolis concentration of 10%, and showed some decline at higher propolis concentrations. At 15 °C, though there were some variations, the inhibition also increased with increasing propolis concentrations, reaching the highest level at the concentration of 25% of propolis. The pairwise post-hoc *T*-test results are shown in Supplementary Tables S1 and S2.

### 2.2. Suppression of Germination of Pd Spores 

**Figure 2:**
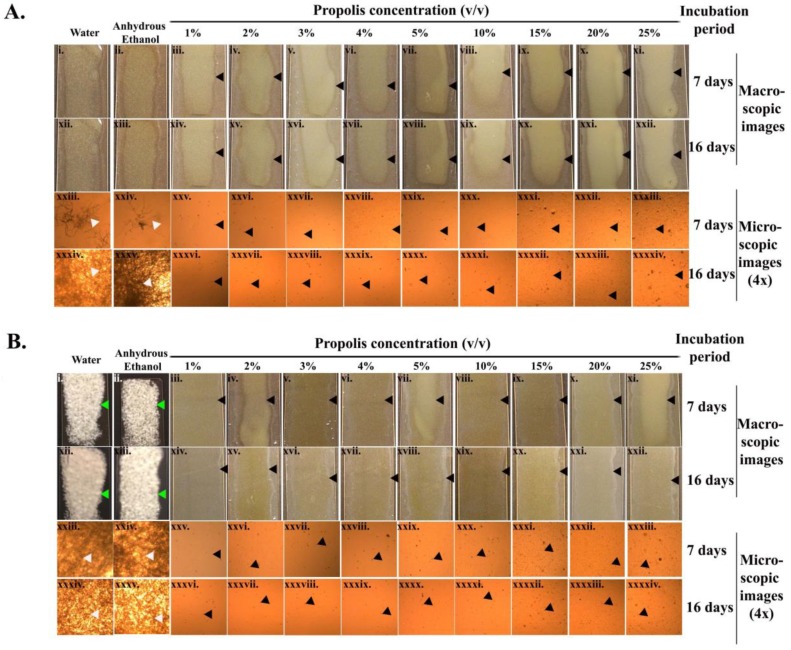
Germule suppression assay. (**A**,**B**) represent the *Pd* germination assay for treatments with water, anhydrous ethanol, and various concentrations of propolis at 8 °C and 15 °C, respectively. The white arrowheads indicate the mycelial extension of the *Pd* spores at two different incubation temperatures. The black arrowheads indicate the inhibition of the *Pd* spores on exposure to propolis at different concentrations. The green arrowheads indicate the formation of white *Pd* lawns resulting from the treatment of spores with water or anhydrous ethanol.

As revealed by the macroscopic and microscopic assay images (Figure 2A,B), there was a complete inhibition of *Pd* sporulation with all propolis concentrations throughout the entire incubation period (16 days) at both incubation temperatures tested. On the seventh day of incubation at 8 °C, more mycelial extensions (4× microscopic images, Figure 2(Axxiii,xxiv)) were observed in the samples treated with water or anhydrous ethanol. The mycelial growth became more confluent amongst the *Pd* spores treated with water and ethanol by the 16th day of incubation (Figure 2(Axxxiv,xxxv)). At 15 °C, the *Pd* spores treated with water and ethanol exhibited a confluent growth from the seventh day of incubation (Figure 2(Bi,ii,xxiii,xxiv)).

### 2.3. Microscopic Examination of the Treated Pd Spores

**Figure 3:**
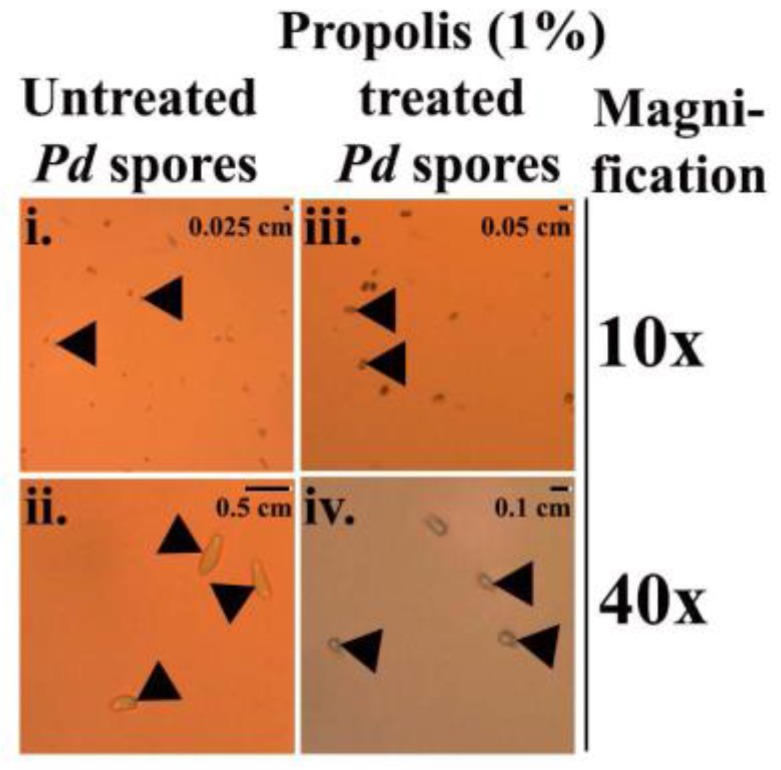
Micrographs of *Pd* spores displayed at 10× and 40× magnification: (**i**–**ii**) elliptical shape of untreated *Pd* spores; (**iii**–**iv**) deformed *Pd* spores treated with 1% propolis.

*Pd* spores treated with 1% propolis revealed a complete deformation (Microscopic images at 10× and 40×, Figure 3(iii,iv)) in vitro when compared to the untreated spores that exhibited elliptical shapes, typical of *Pd* spores. (Figure 3(i,ii)).

### 2.4. Chemical Composition of Propolis

**Figure 4:**
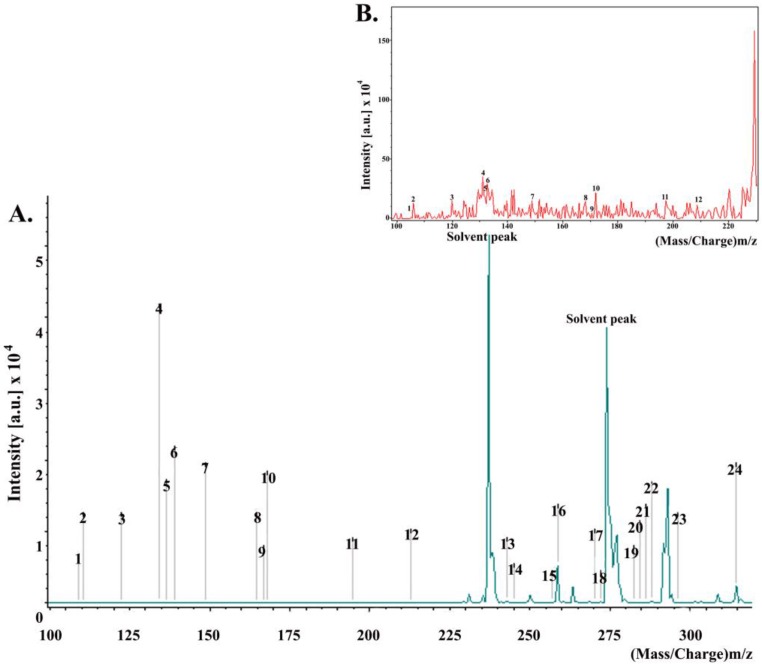
(**A**) MALDI-TOF-MS of a propolis sample at a mass range of 100–400 Da. Each of the peaks on the mass spectrum represents a distinctive compound in our propolis sample. The numbers above the peaks correspond to the compounds listed in Table 1; (**B**) magnified version of the mass spectrum at a mass range of the 100–225 Da.

MALDI spectra (Figure 4) revealed that the major constituents of the propolis used in this study were aromatic acids, i.e., cinnamic acid and p-coumaric acid; dihydrochalcones, i.e., 2,4,6-trihydroxydihydrochalcone; fatty acids, i.e., stearic acid, palmitic acid; esters, i.e., benzyl methoxybenzoate.

## 3. Discussion

The populations of hibernating bats in North America are declining at unprecedented rates because of WNS [1,37]. Because insectivorous bats usually eat insects, they play important roles in the ecosystem and provide valuable pest control services to the agricultural and forestry sectors of the North American economy [11]. Our research has identified a new potential tool for combatting WNS that is threatening many bats across the continent. We have discovered that bee propolis may be used as an effective antifungal agent against *Pd*, the causative agent of WNS.

Our study has revealed that even low concentration (1%) of commercially available propolis (65% tincture) can completely inhibit *Pd* spore germination at both 8 °C and 15 °C. Unfortunately, *Pd* grows optimally between 8–16 °C [3], and the hibernating environment creates an ideal condition for Pd growth on hibernating bats. The incubation temperatures used in our testing were thus representative of the effective hibernation and fungal infection range that are also found in cave environments [38]. Additionally, the bioassay plates were kept until the 60th day after the 22nd day of observation, and we never found any encroachment of growth of *Pd* spores in the observed ZOI. This may indicate that propolis can completely inhibit *Pd* spore germination for up to 60 days when tested in the lab setting.

Propolis has previously been shown to exhibit antifungal, antibacterial, and antiviral properties and therefore has been widely used in human healthcare for treating ulcers, wounds, and rheumatisms [27,28]. We employed the Kirby–Bauer diffusion assay to identify fungicidal activities of propolis, while the germule suppression assay was performed in order to understand the possible inhibitory mechanism of the propolis against *Pd* spores germination. Similar studies by Cornelison el al. 2014 [13,39] have shown that bacteria-derived volatile compounds that include decanal, 2-ethyl-hexanol, nonanal, benzothiole, and N, N-dimethyloctylamine, completely inhibited the growth of conidia and radial mycelial extensions. Moreover, our findings show a deformation of the *Pd* spores exposed to propolis, a result consistent with a possible fungicidal mechanism of action. The deformation of the fungal conidia by propolis has never been reported before.

Though the propolis used in this study was purchased from a western Canadian company, Natural Factors, the company obtained the raw propolis materials from a variety of sources and geographic regions. Further examinations of our purchased propolis traced its origin to Mongolia. Unfortunately, no further information could be obtained, including the specific region within Mongolia, or the plants that bees were feeding on (personal communication). However, consistent with previous findings [40,41,42], our MALDI-TOF analysis of the tested propolis identified a range of constituents. These included benzyl benzoate, benzyl methoxybenzoate, benzyl dihydroxybenzoate, hydroxyacetophenone, 2,4,6-trihydroxydihydrochalcone, pinostrobin chalcone, 2,6-dihydroxy-4-methoxydihydrochalcone, 2,4,6-trihydroxy-4-methoxydihydrochalcone, cinnamic acid, and p-coumaric acid as major constituents. A previous study of Canadian propolis from two regions showed that the propolis from Victoria contained mainly p-hydroxyacetophenone, benzyl hydroxybenzoate, cinnamic acid, and dihydrochalcones, while that from Richmond had large amounts of cinnamic acid and p-coumaric acid [40]. However, both propolis samples showed significant antioxidant properties with a high level of radical scavenging activity. The other remaining compounds identified in our propolis also showed some geographic specificity in previous studies. For example, 3,3-dimethylallyl caffeate was reported from European poplar-type propolis [41,42], while we identified cinnamyl caffeate in our propolis. Other compounds from diverse locations include hydroquinone (Burdock et al. 1998 [42]) and benzyl alcohol [43]. Benzyl alcohol has anti-inflammatory, antibacterial, antitumour, hepatoprotective, and antioxidant activities [41]. Benzoic acid and 4-hydroxybenoic acid found in our samples were previously found in an Iranian propolis, and have shown antibacterial properties [44]. Other constituents revealed in our propolis included ferulic acid, oleic acid, stearic acid, palmitic acid, and pinobanksin 3-O-acetate. An earlier analysis of Anatolian propolis also identified the above mentioned compounds and showed that they exhibited antibacterial activities against Gram-positive bacteria such as *Staphylococcus aureus* (6538-P), *Streptococcus sobrinus*, *Staphylococcus epidermidis*, *Streptococcus mutans*, *Enterococcus faecalis*, and *Micrococcus luteus* [45]. Certain Gram-negative bacteria, such as *Escherichia coli*, *Salmonella typhimurium*, *Pseudomonas aeruginosa*, and *Enterobacter aerogenes* and yeast such as *Candida albicans*, *C. tropicalis*, and *C. krusei* were reported to be susceptible to the Anatolian propolis [45]. Lastly, Sakuranetin, one of the flavonoids identified in our propolis, was reported to exhibit antimicrobial activities against oral pathogens [46].

We have confirmed the complete inhibition of *Pd* spore germination even at a low concentration of propolis (1%). Propolis, also called “bee glue” [21,47], is soluble in anhydrous ethanol, which eliminates the resinous and sticky properties of this substance making it suitable for application on roost substrates. Our study contributes to a growing portfolio of biological and chemical measures for controlling the growth of *Pd* [10,12,13,42]. Future applications on bats, and tests in wild hibernacula are required to test the effectiveness of propolis outside of a laboratory setting.

The US Fish and Wildlife Service (USFWS) has also recommended a number of decontaminants. The only appropriate methods for laboratory and field decontamination of equipment and clothing include ethanol (≥60%), isopropanol (≥60%), isopropyl alcohol wipes (70%), hydrogen peroxide wipes (3%), Accel^®^, Clorox^®^ bleach, Clorox^®^ wipes, Clorox^®^ Clean-Up cleaner + bleach, Hibiclens^®^, and Lysol^®^ IC quaternary disinfectant cleaner. Whether propolis would be useful as a decontamination substance is yet to be seen, in any case the substances listed in the current US national WNS decontamination protocol are more readily available. The toxicity of many of these listed decontamination substances is of minor concern for an equipment decontamination protocol, but it is of utmost concern when these substances are employed for the mitigation of a disease. Naturally sourced anti-*Pd* substances like propolis could provide treatment options that are generally considered safe for mammals. Previous studies reported that Greek and Roman physicians prescribed propolis as a mouth disinfectant and for the topical therapy of cutaneous and mucosal wounds in humans [16]. More recently, a study showed that propolis paste applied on dogs’ cutaneous wounds resulted in better wound re-epithelization, contraction, and total wound healing than a placebo [48]. However, no study has examined the effects of propolis on bats.

Overall, our study has demonstrated the complete inhibition of *Pd* spore germination by propolis. However, significant research is still required, for example an investigation on whether a longer period of testing time in the laboratory would yield any additional results further indicating a real potential of propolis as one of the treatment options for WNS. The potential activities of the individual constituents of propolis and their combinations, as well as their synergistic interactions against *Pd* spore germination and mycelial growth also need to be identified. At present, the medicinal benefits of propolis have been attributed to its complex composition and to the potential synergistic effects of its chemical constituents. In addition, the chemical composition of propolis is highly dependent on its geographical origin, on bee species, and on the botanical food sources of the bees. Further investigations are needed in order to determine whether propolis, or its individual ingredients or combinations thereof may be an option for the treatment of *Pd*-infected cave or bats.

## 4. Materials and Methods

### 4.1. Cultivation of Pd Spores

The *P. destructans* M3906-2 strain was used in this study. This *Pd* strain was previously described by Khankhet et al. (2014) [49]. The cultivation and isolation of the *Pd* spores were performed as previously described [37]. *Pd* cultures were maintained on Sabouraud dextrose agar (SDA) plates at 15 °C. *Pd* spores were isolated from cultures by submerging the conidial lawn in Conidia Harvesting Solution (CHS) (0.05% Tween 80, 0.9% NaCl) for 5 min followed by mechanical scrapping and filtration through glass wool as previously described [13,14]. The concentration of the *Pd* spores was quantified by a haemocytometer and the spores were stored in phosphate buffer saline (PBS) at 4 °C until further use.

### 4.2. Kirby–Bauer Diffusion Assay

One hundred microliter of isolated *Pd* spores (1 × 10^7^ spores/mL) were mixed with 250 mL of Sabouraud dextrose agar (SDA) media supplemented with chloramphenicol (34 mg/L) at 50 °C (Fisher Scientific, Fairlawn, NJ, USA) to avoid bacterial contamination. Approximately 20–22 mL of the mixture was poured into each of the 85 mm petri plates. Plates were air-dried in a laminar airflow hood. Eight millimeter (diameter) paper discs (Toyo Roshi Kaisha Ltd., Tokyo, Japan) were soaked in different concentrations (1%, 2%, 3%, 4%, 5%, 10%, 15%, 20%, and 25%) of commercially available propolis (65% extract) (Natural Factors, Coquitlam, BC, Canada), air-dried, and placed in the center of each seeded SDA medium plate along with sterile water and anhydrous ethanol (<0.005% water) (Sigma Aldrich, St. Louis, MO, USA); the latter two conditions were used as controls. Anhydrous ethanol was tested since it was used as a solvent to dissolve propolis in all dilutions (as per the manufacturer’s instructions). All plates with different propolis concentrations were incubated at 8 °C or 15 °C, including control plates, in duplicate. Anti-*Pd* activities were identified as zones of inhibition around the impregnated paper discs, and the diameters were measured in millimeters with an electronic Vernier caliper (Guilin, Guangxi, China). Notably, the measurement of the diameters for the zones of inhibition were recorded on the 22nd and 15th day of incubation at 8 °C and 15 °C, respectively, since *Pd* spores germinated slower at 8 °C than at 15 °C. Each treatment was repeated four times. The bioassay plates were kept until the 60th day after the observation performed at the 22nd day.

To determine whether the concentrations of propolis differed in their inhibitory effects, we used a single-factor ANOVA to analyze the quantitative-zone-of-inhibition data. If an overall difference was found, all pairwise comparisons were made using the two-tailed t-test. Since there were 36 pairwise comparisons [9 concentrations × (9 − 1)/2 = 36] at each of the two temperatures, a Bonferroni correction was applied to the typical p value of < 0.05 considered statistically significant. The corrected *p* value was 0.05/36 = 0.0013888.

### 4.3. Germule Suppression Assay

Sterile microscopic slides (24.5 × 76.2 mm) were layered with 600 µL of molten SDA (10%) at a temperature of 50 °C and premixed with 5 µL of *Pd* spores (1 × 10^7^ spores/mL) and 5 µL of each of the indicated concentrations of propolis. Quadruplet slides were prepared for each of the propolis concentrations and were incubated in duplicate at 8 °C and 15 °C. The germination of the *Pd* spores was measured macroscopically by visualizing the white growth confluence of the *Pd* lawn, and microscopically by the *Pd* hyphal extension. Both macroscopic and microscopic images were acquired from slides on the 7th and 16th day of incubation. The microscopic images were taken using a DCM 130E digital camera for microscope (1.3 M pixels, CMOS chip) (AmScope, Irvine, CA, USA). The microscopic images were imported with the Scope Photo Software (AmScope, Irvine, CA, USA).

### 4.4. Analysis of Chemical Constituents

The chemical composition of propolis was determined using matrix-assisted laser desorption ionization time-of-flight mass spectrometry (MALDI-TOF-MS). The propolis sample consisted of 50% diluted propolis in methanol covered with 0.1020 M of α-cyano-4-hydroxy cinnamic acid (HCCA) in 1:4 (*v*/*v*) H_2_O/acetonitrile. A 1.0 µL diluted aliquot of the sample was first spotted on the plate and covered with 2.0 µL of matrix. The mass spectra were obtained using a bench-top Microflex MALDI-TOF MS from Bruker Daltonics^®^ (Bremen, Germany) equipped with a pulsed nitrogen laser at 355 nm wavelength. The spectra were recorded from 40 to 2000 Da (positive mode), and from 100 to 400 Da (positive mode) using FlexControl 3.3 software (ion source 1: 19 kV; ion source 2: 15.5 kV; lens voltage: 9.45 kV; laser frequency: 60 Hz; pulsed ion extraction (PIE) delay: 120 ns). Other parameters are shown in Table 2. Mass gates of 400 *m*/*z* (positive mode) were set for all experiments. Individual mass spectra from each spot were acquired by averaging 350 laser shots. Data acquisition was set to automate, and the “random walk” movement was activated at 10 shots per raster during the sequence. The peak lists and intensities were calculated using the peak-picking centroid algorithm in FlexAnalysis 3.3 software.

## Figures and Tables

**Table 1:** Composition of propolis as determined by MALDI-TOF-MS (the peaks corresponding to these values can be seen in the mass spectrum in Figure 4).

Peak	Constituents Identified	Mass/Charge (*m*/*z*)	Intensity
1	Benzyl alcohol	108.14	0.77
2	Hydroquinone	110.11	5.00
3	Benzoic acid	122.12	5.93
4	Cinnamyl alcohol	134.17	23.81
5	Hydroxyacetophenone	136.15	7.82
6	4-Hydroxybenzoic acid	138.12	10.98
7	Cinnamic acid	148.16	14.81
8	p-coumaric acid	164.16	4.91
9	3-Phenyl-3-hydroxypropanoic acid	166.18	5.91
10	Sesquiterpenes	168.31	13.90
11	Ferulic acid	194.18	3.91
12	Benzyl benzoate	212.25	6.85
13	Benzyl methoxybenzoate	242.27	211.88
14	Benzyl dihydroxybenzoate	244.24	105.16
15	Palmitic acid	256.43	64.16
16	2,4,6-Trihydroxydihydrochalcone	258.27	5184.70
17	Pinostrobin chalcone	270.28	47.98
18	2,6-Dihydroxy-4-methoxydihydrochalcone	272.25	132.69
19	Oleic acid	282.47	28.64
20	Stearic acid	284.31	50.69
21	Sakuranetin	286.27	44.71
22	2,4,6-Trihydroxy-4-methoxydihydrochalcone	288.30	247.59
23	Cinnamyl caffeate	296.32	44.87
24	Pinobanksin 3-O-acetate	314.29	2331.83

**Table 2:** Parameters of MALDI-TOF-MS.

Parameters	Values
Laser	Pulsed nitrogen
Laser power	20–80%
Peak selection (mass range)	40–2000 Da
Sample rate	0.05 GS/s
Mass range	Low range
Electronic gain	Enhanced 100 mV
Realtime smooth	Off
Spectrum size	2069 pts
Spectrum delay	307 pts
Laser frequency	60.0 Hz
Laser attenuator offset	17%
Laser attenuator range	30%
Target	MSP 96 target polished steel
Matrix	α-cyano-4-hydroxy-cinnamic acid, HCCA
Sample	50% diluted propolis in MeOH covered with 0.1020 M of HCCA in 1:4 (*v*/*v*) H_2_O/acetonitrile

## Supplementary Materials

The following are available online at www.mdpi.com/2079-6382/7/1/2/s1, Table S1: Analysis of Variance (ANOVA) One-way (8 °C incubation temperature), Table S2: Analysis of Variance (ANOVA) One way (15 °C incubation temperature).
