# Supplementary material for: Evidence for Anti-Pseudogymnoascus destructans (Pd) Activity of Propolis
Source: Antibiotics (Basel). 2017 Dec 21;7(1):2. doi: 10.3390/antibiotics7010002 (PMC5872113; doi:10.3390/antibiotics7010002)
Supplement: Supplementary file 1 [file antibiotics-07-00002-s001.pdf]

antibiotics

MDPI

Article

Evidence for anti-*Pseudogymnoascus destructans* (*Pd*) Activity of Propolis

Table S1: Analysis of Variance (ANOVA) One way (8 °C incubation temperature) Summary

Concentration of Propolis (%)(Groups)	Sample size	Sum	Mean	Variance
1	4	38.98	9.745	0.0091
2	4	40.84	10.21	0.33127
3	4	42.2	10.55	2.24547
4	4	38.63	9.6575	0.11862
5	4	56.54	14.135	6.1123
10	4	58.99	14.7475	0.29369
15	4	57.09	14.2725	0.73942
20	4	53.07	13.2675	7.53216
25	4	43.43	10.8575	2.12883

ANOVA

Source of Variation	SS	df	MS	F	p-level	F-crit
Between groups	144.10999	8	18.01375	8.30941	0.00001	2.30531
Within groups	58.53258	27	2.16787			
Total	202.64256	35				

Pairwise T-test

	1	2	3	4	5	10	15	20	25
**1**									
**2**	1.59408								
**3**	1.07225	0.42362							
**4**	0.48967	1.64744	1.16093						
**5**	3.5487	3.09248	2.48013	3.58748					
**10**	18.1822	11.47945	5.26836	15.85376	0.484				
**15**	10.4661	7.8522	4.30924	9.96427	0.10506	0.93465			
**20**	2.56542	2.18067	1.73813	2.61026	0.4697	1.0581	0.69888		
**25**	1.52172	0.82565	0.29405	1.60091	2.28339	4.99857	4.03285	1.55073	

P = 0.05/32 = 0.0015625; The values in red indicate that these comparisons are statistically significant.

Table S2: Analysis of Variance (ANOVA) One way (15 °C incubation temperature) Summary

Concentration of Propolis (%)(Groups)	Sample size	Sum	Mean	Variance
1	4	38	9.5	1.2204
2	4	43.34	10.835	0.71737
3	4	46.95	11.7375	2.58543
4	4	43.49	10.8725	0.12202
5	4	50.23	12.5575	0.24016
10	4	50.24	12.56	2.18453
15	4	60.31	15.0775	1.08743
20	4	50.01	12.5025	5.09249
25	4	58.76	14.69	0.1544

ANOVA

Source of Variation	SS	df	MS	F	p-level	F-crit
Between groups	103.7076	8	12.96345	8.70405	8.39E-06	2.30531
Within groups						
Total	40.21268	27	1.48936			

Pairwise T-test

	1	2	3	4	5	10	15	20	25
1									
2	1.91805								
3	2.29387	0.9932							
4	2.36918	0.08186	1.05139						
5	5.05984	3.52058	0.97564	5.59971					
10	3.31663	2.02525	0.7532	2.22224	0.00321				
15	7.34291	6.31594	3.48558	7.64719	4.37421	2.78353			
20	2.39001	1.38361	0.55217	1.42761	0.04763	0.04263	2.07165		
25	8.85274	8.25761	3.56745	14.5218	6.7899	2.78548	0.69546	1.90997	

P = 0.05/32 = 0.0015625; The values in red indicate that these comparisons are statistically significant.

© 2017 by the authors. Submitted for possible open access publication under the terms and conditions of the Creative Commons Attribution (CC BY) license (http://creativecommons.org/licenses/by/4.0/).
